# Estimating effects of rare haplotypes on failure time using a penalized Cox proportional hazards regression model

**DOI:** 10.1186/1471-2156-9-9

**Published:** 2008-01-25

**Authors:** Olga W Souverein, Aeilko H Zwinderman, J Wouter Jukema, Michael WT Tanck

**Affiliations:** 1 Department of Clinical Epidemiology, Biostatistics and Bioinformatics, Academic Medical Center, University of Amsterdam, P.O. Box 22700, 1100 DE, Amsterdam, The Netherlands; 2 Department of Cardiology, Leiden University Medical Center, Leiden, The Netherlands

## Abstract

**Background:**

This paper describes a likelihood approach to model the relation between failure time and haplotypes in studies with unrelated individuals where haplotype phase is unknown, while dealing with the problem of unstable estimates due to rare haplotypes by considering a penalized log-likelihood.

**Results:**

The Cox model presented here incorporates the uncertainty related to the unknown phase of multiple heterozygous individuals as weights. Estimation is performed with an EM algorithm. In the E-step the weights are estimated, and in the M-step the parameter estimates are estimated by maximizing the expectation of the joint log-likelihood, and the baseline hazard function and haplotype frequencies are calculated. These steps are iterated until the parameter estimates converge. Two penalty functions are considered, namely the ridge penalty and a difference penalty, which is based on the assumption that similar haplotypes show similar effects.

Simulations were conducted to investigate properties of the method, and the association between *IL10 *haplotypes and risk of target vessel revascularization was investigated in 2653 patients from the GENDER study.

**Conclusion:**

Results from simulations and real data show that the penalized log-likelihood approach produces valid results, indicating that this method is of interest when studying the association between rare haplotypes and failure time in studies of unrelated individuals.

## Background

In recent years there has been a great interest in associating haplotypes with complex disease phenotypes, and many statistical models have been described. These models are complicated by individuals that are heterozygous on two or more of these SNPs, because their haplotypes cannot be determined with certainty. Consider for instance two SNPs with alleles *A *or *a*, and *B *or *b*. Individuals that are heterozygous for both SNPs, have genotypes *Aa *and *Bb*, and they inherited either haplotype *AB *from one parent and *ab *from the other, or they inherited haplotypes *Ab *and *aB*. Hence, it is unknown whether these individuals have haplotype pair *AB/ab *or haplotype pair *Ab/aB*. This uncertainty complicates statistical inference and if the number of biallelic single nucleotide polymorphisms (SNPs) is large, or when allele frequencies of the SNPs are close to 50%, many individuals will be multiple heterozygous.

Most of the current methods focus on continuous or dichotomous outcome data [1-9], while only few can be applied in cohort studies [10,11]. Another concern is related to the presence of rare haplotypes, which is a very common problem in genetic association studies. In the present paper we adopt the suggestion of Tanck et al. [12] to use a weighted penalized likelihood method to estimate the association between a phenotype and the set of haplotypes, which may include rare haplotypes. We consider a model for the relation between a failure time *T *measured in *N *unrelated individuals and the haplotypes of these individuals formed by *m *SNPs measured in a single gene. Previously, Lin [10] has described a similar method for haplotype analysis in cohort studies, but this method did not include a penalty function for dealing with the unstable estimates of rare haplotypes.

In the sequel we will first describe the kind of data that we analyze, then we will described our statistical model, and the algorithm to estimate the parameters of our model. We will use simulated data to illustrate some characteristics of the estimators, and finally we will analyze real data from the GENDER study on cardiovascular disease [13].

## Results

### Algorithm

#### Data and model

We consider a sample of *i *= 1,..., *N *unrelated individuals with failure- or censored-time *t*_*i*_. The indicator *d*_*i *_is used to indicate whether *t*_*i *_is an event-time (*d*_*i *_= 1), or a censored-time (*d*_*i *_= 0). Let *g*_*i *_be a vector of *m *biallelic SNPs measured in individual *i*. With *m *biallelic SNPs there are *j *= 1,.., *Nhap *= 2^*m *^different haplotypes possible with population frequencies *p*_1_,..., *p*_*j*_,... *p*_*Nhap*_.

Suppose all haplotypes were observed in all patients, then these could be represented with the vector *x*_*i *_of length *Nhap*, where *x*_*ij *_equals 0, 1, or 2, depending on the number of haplotypes of type *j *observed in patient *i*. (Notice that ∑_*j*_*x*_*ij *_= 2, meaning that only *Nhap *- 1 contrasts are identifiable.) The conditional hazard function for failure at *t*_*i *_given *x*_*i *_can then be specified as

*ln*(*h*(*t*_*i*_|*x*_*i*_)) = *ln*(*h*_0_(*t*_*i*_)) + *β'x*_*i*_,

where *h*_0_(*t*) is an unspecified baseline hazard function, and *β *a vector of regression parameters. The survival function *S*(*t*_*i*_|*x*_*i*_) equals

$$S(t_{i}|x_{i})=e^{-H_{0}(t_{i})e^{\beta^{\prime }x_{i}}},$$

where *H*_0_(*t*) is the unspecified baseline cumulative hazard function.

In our case, haplotypes were not observed in all patients, and therefore we model the survival function conditional on the observed genotypes as a weighted mixture over all *z*_*i *_possible haplotype pairs given genotypes *g*_*i*_:

$$S(t_{i}|g_{i})=\sum_{q=1}^{z_{i}}w_{iq}S_{q}(t_{i}|x_{iq})$$

where *S*_*q*_(*t*_*i*_|*x*_*iq*_) is the survival function specified as in equation (2), and *x*_*iq *_is the *q*^*th *^haplotype pair that is possible given genotypes *g*_*i*_, and *w*_*iq *_is the probability that individual *i *has haplotype pair *q *given genotypes *g*_*i*_: *w*_*iq *_= *Pr*(*X*_*i *_= *x*_*iq*_|*g*_*i*_).

In most circumstances the population haplotype frequencies *p*_1_,..., *p*_*Nhap *_are unknown, and must be estimated from the data at hand. But suppose these are known, then under the assumption of Hardy-Weinberg equilibrium the probability that an individual is carrying haplotype pair *q *= (*h, r*) is calculated as ph * *pr *(*h*, *r *= 1,..., *Nhap*). Consequently, when observing genotype vector *g*_*i *_the probability that individual *i *has haplotype pair *q *= (*h*, *r*) equals:

$$w_{iq}=\frac{p_{h}p_{r}d_{hri}}{\sum_{h=1}^{Nhap}\sum_{r=1}^{Nhap}p_{h}p_{r}d_{hri}}$$

where the summation takes place over all haplotype pairs that are compatible with genotypes *g*_*i *_and *d*_*hri *_is an indicator function, which = 1 when the haplotype pair (*h*, *r*) is compatible with *g*_*i *_and 0 otherwise.

Given the weights *w*_*iq *_the full likelihood of the data given the model in equation (3) equals

$$L=\prod_{i=1}^{N}S(t_{i}|g_{i}){\left(\frac{-\partial ln(S(t_{i}|g_{i}))}{\partial t}\right)}^{d_{i}}=\prod_{i=1}^{N}\sum_{q=1}^{z_{i}}w_{iq}S_{q}(t_{i}|x_{iq}){\left(h_{0}(t_{i})e^{\beta^{\prime }x_{iq}}\right)}^{d_{i}},$$

and the regression parameters *β*, and the baseline cumulative hazard function *H*_0_(*t*) may be estimated by maximizing (5). If *H*_0_(*t*) is a parametric function, maximization of (5) is uncomplicated. If *H*_0_(*t*) is a nonparametric function, we follow a similar argument as Breslow [14]. If events occurred at times *τ*_1_,..., *τ*_*j*_,..., *τ*_*k*_, we shift all censored observations in the interval [*τ*_*j*-1_, *τ*_*j*_) to *τ*_*j*-1_, and assume (piecewise) constant hazard in the intervals. In that case the logarithm of the likelihood (5) reduces to

$$lnL^{Breslow}=\sum_{j=1}^{k}ln(\lambda_{j})+\sum_{i=1}^{N}ln\left(\sum_{q=1}^{z_{i}}w_{iq}e^{-e^{\beta^{\prime }x_{iq}}\sum_{j=1}^{k}\lambda_{j}(\tau_{j+1}-\tau_{j})I(t_{i}>\tau_{j})}e^{d_{i}\beta^{\prime }x_{iq}}\right),$$

where *λ*_*j *_is the jump in the cumulative baseline hazard function at time *τ*_*j*_, and *I*(*τ*_*i *_> *τ*_*j*_) is an indicator function.

Estimation equations for *H*_0_(*t*) and *β *can be derived by equating to zero the first-order derivatives of the log-likelihood in (6). For *λ*_*j *_we find

$$\lambda_{j}=\frac{1}{(\tau_{j+1}-\tau_{j})\sum_{i=1}^{N}\left(I(t_{i}>\tau_{j})\sum_{q=1}^{z_{i}}{\tilde{w}}_{iqt_{i}}e^{\beta^{\prime }x_{iq}}\right)},$$

and for *β*_ℓ _(ℓ = 1,..., *Nhap*):

$$\sum_{i=1}^{N}\sum_{q=1}^{z_{i}}{\tilde{w}}_{iqt_{i}}X_{iq\ell }\left(d_{i}-e^{\beta x_{iq}}\sum_{j=1}^{k}\lambda_{j}(\tau_{j+1}-\tau_{j})I(t_{i}>\tau_{j})\right)=0,$$

where

$${\tilde{w}}_{iqt_{i}}=\frac{w_{iq}S_{q}(t_{i}|x_{iq})e^{d_{i}\beta^{\prime }x_{iq}}}{\sum_{q=1}^{z_{i}}w_{iq}S_{q}(t_{i}|x_{iq})e^{d_{i}\beta^{\prime }x_{iq}}}.$$

Notice that if *z*_*i *_= 1 for all *i*, thus when all haplotypes were observed, then ${\tilde{w}}_{iqt_{i}}$ = 1, and equation (7) reduces to the usual Breslow estimator of *H*_0_(*t*), and equation (8) to the usual Cox estimator of *β*.

Unfortunately, the weights ${\tilde{w}}_{iqt_{i}}$ depend on both *H*_0_(*t*) and *β*.

#### Estimation algorithm

Since in practice the population haplotype frequencies *p *must be estimated together with *β *and *H*_0_(*t*), and because ${\tilde{w}}_{iqt_{i}}$ depends on *H*_0_(*t*) and *β*, direct maximization of (5) or (6) is complicated. Instead we propose to use an EM algorithm. For that we consider the covariate vector *x*_*i *_as missing, and we maximize the expectation of the joint log-likelihood of *L*((*t*_*i*_, *d*_*i*_), *x*_*i*_|*g*_*i*_) over the posterior probabilities of *x*_*i *_given the observed genotypes *g*_*i*_, and given the observed failure/censoring times (*t*_*i*_, *d*_*i*_) and given current estimates of the parameters *β*, *H*_0_(*t*), and *w*_*iq*_:

$$\begin{array}{l} \sum_{i=1}^{N}E[lnL((t_{i},d_{i}),x_{i}|g_{i},\beta^{\left(a\right)},H_{0}{\left(t\right)}^{\left(a\right)},w_{iq}^{\left(a\right)})]=\\ \;\sum_{i=1}^{N}\sum_{q=1}^{z_{i}}{\hat{w}}_{iqt_{i}}\left[d_{i}ln(h_{0}(t_{i}))+d_{i}\beta^{\prime }x_{iq}-H_{0}(t_{i})e^{\beta^{\prime }x_{iq}}+ln(w_{iq})\right] \end{array}$$

where ${\tilde{w}}_{iqt_{i}}$ is the posterior probability that *X*_*i *_= *x*_*iq *_given ((*t*_*i*_, *d*_*i*_), *g*_*i*_, *β*^(*a*)^, *H*_0_(*t*)^(*a*)^, $w_{iq}^{\left(a\right)}$:

$$P(X_{i}=x_{iq}|g_{i},(t_{i},d_{i}),\beta^{\left(a\right)},H_{0}{\left(t\right)}^{\left(a\right)},p^{\left(a\right)})=\frac{e^{d_{i}\beta^{\left(a\right)}x_{iq}-H_{0}{\left(t_{i}\right)}^{\left(a\right)}e^{\beta^{\left(a\right)}x_{iq}}}w_{iq}^{\left(a\right)}}{\sum_{r=1}^{z_{i}}e^{d_{i}\beta^{\left(a\right)}x_{ir}-H_{0}{\left(t_{i}\right)}^{\left(a\right)}e^{\beta^{\left(a\right)}x_{ir}}}w_{ir}^{\left(a\right)}},$$

and

$$w_{iq}^{\left(a\right)}=\frac{p_{h}^{\left(a\right)}p_{r}^{\left(a\right)}d_{hri}}{\sum_{h=1}^{Nhap}\sum_{r=1}^{Nhap}p_{h}^{\left(a\right)}p_{r}^{\left(a\right)}d_{hri}}$$

The EM algorithm consists then of iterating two steps. In the M-step of iteration *a *+ 1, (*β*, *H*_0_(*t*), *w*_*iq *_= *p*_*j*_*p*_*l*_*/Σp*_*r*_*p*_*s*_) are estimated by maximizing (10) given ${\tilde{w}}_{iqt_{i}}$ evaluated using (*β*^(*a*)^, *H*_0_(*t*)^(*a*)^, $w_{iq}^{\left(a\right)}$), and in the E-step, ${\tilde{w}}_{iqt_{i}}$ is re-estimated given using (11) with (*β*^(*a*+1)^, *H*_0_(*t*)^(*a*+1)^, $w_{iq}^{\left(a+1\right)}$). Estimation equations of *β *and *H*_0_(*t*) are the same as in (8) and (7), but $\tilde{w}$ replaced with $\hat{w}$, and these are solved iteratively. The haplotype relative frequencies *p*_*j *_are estimated as

$$p_{j}^{\left(a+1\right)}=\frac{1}{2N}\sum_{i=1}^{N}\sum_{q=1}^{z_{i}}I(j,q){\hat{w}}_{iqt_{i}},$$

where *I*(*j, q*) is an indicator-function denoting whether haplotype *j *is part of haplotype-combination *q*. Standard errors of *β *can be derived from the information-matrix of the log-likelihood in equation (6):

$$\begin{array}{l} -\frac{\partial^{2}lnL^{Breslow}}{\partial \beta_{a}\partial \beta_{b}}=\sum_{i=1}^{n}\sum_{q=1}^{z_{i}}X_{iqa}X_{iqb}{\hat{w}}_{iqt_{i}}H_{0}(t_{i})e^{\beta x_{iq}}-\\ \sum_{i=1}^{n}\sum_{q=1}^{z_{i}}X_{iqa}X_{iqb}{\hat{w}}_{iqt_{i}}{\left(d_{i}-H_{0}(t_{i})e^{\beta x_{iq}}\right)}^{2}+\\ \sum_{i=1}^{n}(\sum_{q=1}^{z_{i}}{\hat{w}}_{iqt_{i}}X_{iqa}(d_{i}-H_{0}(t_{i})e^{\beta x_{iq}}))(\sum_{q=1}^{z_{i}}{\hat{w}}_{iqt_{i}}X_{iqb}(d_{i}-H_{0}(t_{i})e^{\beta x_{iq}})) \end{array}$$

Since we are mainly interested in the uncertainty of *β*, we only used that part of the hessian that pertains to *β*. Notice that the first term of (14) equals the hessian-matrix that is used in the M-step of the EM algorithm.

#### Penalized log-likelihood

Mutations in general tend to be rare, and so are the haplotypes in which they are encompassed. Furthermore, when ten loci are considered there are 2^10 ^= 1024 different haplotypes possible, many of which will have low frequency in samples up to thousands of individuals. If haplotypes have low frequency their associated hazard ratio estimate will be unstable. We used a penalized log-likelihood method to obtain more stable parameter estimates. Basically, we optimized the penalized log-likelihood ℓ^*p*^, defined as $\ell^{p}=lnL^{Breslow}-\frac{\lambda }{2}Pen(\beta )$, where *Pen*(*β*) is the penalty function.

As penalty functions we considered the well-known ridge-penalty function $\left(Pen(\beta )=\sum_{a}\beta_{a}^{2}\right)$, and a difference-penalty function (*Pen*(*β*) = ∑_*a*_∑_*b*_*a*_*ab*_(*β*_*a *_- *β*_*b*_)^2 ^(*a *> *b*)), where *a*_*ab *_is a fixed and known value representing the similarity of haplotypes *a *and *b*. We quantified the similarity between haplotypes (*a*_*ab*_) by counting the number of shared alleles which – with *m *loci – varies between zero and *m *- 1.

The penalty parameter *λ *was found by optimizing the cross-validated log-likelihood (CVL) as described by Verweij and van Houwelingen [15]: *CVL*(*λ*) = *lnL*^*Breslow*^(*β*^*λ*^) - *c*(*λ*), where *lnL*^*Breslow*^(*β*^*λ*^) is the log-likelihood evaluated with the penalized log-likelihood estimate *β*^*λ*^. The factor *c*(*λ*) is an approximation of the effective dimension of the model, which in our case depends on the log hazard ratios *β*, the baseline hazard function *H*_0_(*t*), and the haplotype frequencies *p*. For convenience sake, we approximated the effective dimension in the same manner as Verweij and van Houwelingen [15] as

$$c(\lambda )=trace\left([H^{\lambda }(\beta^{\lambda })){]}^{-1}\sum_{i}U_{i}(\beta^{\lambda })U_{i}{\left(\beta^{\lambda }\right)}^{T}\right)$$

where *U*_*i*_(*β*^*λ*^) is the contribution of individual *i *to the first-order derivative of the unpenalized log-likelihood, and *H*^*λ *^(*β*^*λ*^) is the matrix of second-order derivatives of the penalized log-likelihood evaluated at *β*^*λ*^, *H*_0_(*t*), and *p*. Notice that the last term of (15) is equal to the third term of (14).

Although the penalized likelihood estimates of *β *are somewhat biased, and it is therefore somewhat unclear how to interpret standard errors, we nevertheless assessed the stability of the penalized estimates by a parametric bootstrap procedure. We took 200 bootstrap samples, estimated *λ *in each sample by optimizing CVL, and derived standard errors from the distribution of the associated penalized estimates of *β*, *p*, and *H*_0_(*t*). The number of bootstrap samples used was based on results from simulations, in which the number of bootstrap samples varied from 10 to 1000. These data showed that SE estimates were relatively stable after 100 to 200 bootstrap samples.

The EM algorithm presented in this paper was programmed in MATLAB^® ^R 7.0 (The MathWorks, Natick, MA, USA) as well as in a set of R-functions and is freely available upon request from the corresponding author.

### Testing

To illustrate some characteristics of our approach, simulations were carried out. In each replicate, a data set of 200 (simulation 1 and 2) or 2000 (simulation 3) individuals was created in whom 3 loci were measured. We simulated the 8 haplotypes (*x*_1_,..., *x*_8_) to have frequencies of: *p*_000 _= 0.62, *p*_001 _= 0.05, *p*_010 _= 0.02, *p*_011 _= 0.005, *p*_100 _= 0.02, *p*_101 _= 0.003, *p*_110 _= 0.002 and *p*_111 _= 0.28. Given the haplotypes drawn for a specific individual *i*, the survival time S was drawn from the exponential distribution with log(intensity) equal to ∑_*j*_*β*_*j*_*x*_*ij*_. A censoring time C was independently drawn from an independent log-normal distribution such that in about 25% of all individuals *C *<*S*, in which case the survival time was censored at *C*. In each replicate, the haplotype effects were estimated using three models: 1) unpenalized (similar to [10] and [11]), 2) ridge penalized and 3) difference penalized. The statistical properties were evaluated using three different measures, namely the mean bias of the parameter estimates, the mean SE and the coverage probability, which is defined as the probability that the 95% confidence interval of the parameter estimate contains the true theoretical value of the parameter estimate.

Furthermore, for each haplotype the percentage of replicates which identified the haplotype as being significantly associated with the outcome (i.e., power or Type I error rate) was calculated. The significance level used to calculate the power and the Type I error rate was set to *α *= 0.05. In addition, the effect of omitting rare haplotypes (011, 101 and 110) or fixing their effect to the close haplotype 111 on the effect estimates was investigated in the unpenalized models only. For simulation 1 and 2, 500 replicates were carried out, whereas 100 replicates were carried out in simulation 3.

In the first simulation, replicates contained 200 individuals and the haplotypes with a rare allele at locus 2 (010, 011, 110 and 111) were simulated to have a relative risk of 2 compared to the most frequent haplotype 000, corresponding with a regression parameter of *β *= 0.69. This simulation mimics a setting in which the observed effects are due to a single SNP. The observed genotype counts of a random replicate are given in Table 1. In total, 75 (37.5%) individuals were heterozygous on multiple loci of which 63 were heterozygous at all three loci. In 80% of the replicates, all eight haplotypes were present. The mean bias, mean SE, coverage probability and percentage estimates with a p < 0.05 for this simulation based on the 80% 'complete' replicates are shown in Table 2. For the less frequent haplotypes (011, 110 and 101), the introduction of the ridge or difference penalty leads to a substantial reduction in the mean SE as compared to the non-penalized model. On the other hand, the mean SE of the more frequent haplotypes increases somewhat in the penalized models. The effect on the power to detect the effects of the haplotypes 010, 011, 110 and 111 as well as on the type I error probabilities of the haplotypes 001, 100 and 101 is less univocal. In the second simulation, replicates also contained 200 individuals and the haplotypes 001 and 101 were simulated to have a relative risk of 3 (*β *= 1.10) compared to the reference haplotype 000. This simulation mimics a setting in which a particular allele combination on loci 2 and 3 (i.e. ×01) is related to an increased risk. In 85% of the replicates, all eight haplotypes were available. The mean bias, mean SE, coverage probability and percentage estimates with a p < 0.05 for this simulation based on the 85% 'complete' replicates are shown in Table 3. Similar to simulation 1, inclusion of a penalty leads to a large reduction in mean SE of the less frequent haplotypes. The effect of haplotype 101 is estimated more precisely, but the power to find a significant effect for this haplotype is reduced from 25% in the non-penalized model to a value similar to the type I error probability of the haplotypes without an effect in both penalized models. The effect of introducing a penalty on the mean bias, mean SE and power to detect an significant effect of the more frequent haplotype 001 are only minor.

**Table 1:** Numbers of individuals with the various genotypes on three loci in a simulation of 200 individuals

		Locus 3		
Locus 1	Locus 2	wild type^*a*^	heterozygote	homozygote

wild type	wild type	83	13	1
	heterozygote	6	2	0
	homozygote	0	0	0
heterozygote	wild type	6	0	0
	heterozygote	0	63	3
	homozygote	0	4	2
homozygote	wild type	0	0	0
	heterozygote	0	3	1
	homozygote	0	0	13

a) wild type = homozygous for the most frequent allele

**Table 2:** Realized *β*^*a*^, mean bias, mean standard error (SE), coverage probability and percentage of the effects that had a p-value < 0.05 in the simulation where haplotypes with a rare allele at locus 2 had a modeled parameter estimate of 0.69. Each replicate contained 200 individuals

No penalty						
Haplotype	Frequency	Realized^*a*^	Mean bias	Mean SE	Coverage	*P *< 0.05^*b*^
010	0.020	0.741	0.014	0.44	0.95	0.43
011	0.005	0.593	-0.663	2971.81	0.95	0.21
110	0.002	0.000	-0.971	14514.13	0.96	0.10
111	0.280	0.713	0.018	0.09	0.79	1.00
001	0.050	0.012	0.050	0.28	0.91	0.09
100	0.020	0.038	0.010	33.23	0.95	0.05
101	0.003	0.000	-2.212	7674.81	0.90	0.10

Ridge penalty						
Haplotype	Frequency	Realized^*a*^	Mean bias	Mean SE	Coverage	*P *< 0.05^*b*^

010	0.020	0.741	-0.135	0.73	0.85	0.18
011	0.005	0.593	-0.359	1.91	0.74	0.03
110	0.002	0.000	-0.557	1.00	0.39	0.19
111	0.280	0.713	-0.010	0.11	0.83	1.00
001	0.050	0.012	0.037	0.24	0.87	0.13
100	0.020	0.038	0.070	1.08	1.00	0.00
101	0.003	0.000	-0.649	2.16	0.89	0.11

Difference penalty						
Haplotype	Frequency	Realized^*a*^	Mean bias	Mean SE	Coverage	*P *< 0.05^*b*^

010	0.020	0.741	-0.008	0.62	0.98	0.35
011	0.005	0.593	-0.132	1.39	1.00	0.03
110	0.002	0.000	-0.272	1.00	1.00	0.15
111	0.280	0.713	0.008	0.12	0.89	1.00
001	0.050	0.012	0.122	0.24	0.79	0.21
100	0.020	0.038	0.131	0.88	0.93	0.07
101	0.003	0.000	-0.301	1.68	0.91	0.09

a) "Realized" denotes to the median estimate from the simulated data, where the haplotypes were known, and could be used in the Cox model directly; b) For haplotypes 010, 011, 110 and 111 these percentages reflect power, whereas the percentages for the other three haplotypes reflect type I error probabilities.

**Table 3:** Realized *β*^*a*^, mean bias, mean standard error (SE), coverage probability and percentage of the effects that had a p-value < 0.05 in the simulation where haplotypes 001 and 101 had a modeled parameter estimate of 1.10. Each replicate contained 200 individuals.

No penalty						
Haplotype	Frequency	Realized^*a*^	Mean bias	Mean SE	Coverage	*P *< 0.05^*b*^
001	0.050	1.151	0.067	0.25	0.90	0.98
101	0.003	0.817	-0.868	8132.96	0.97	0.25
010	0.020	0.031	0.079	0.48	0.90	0.10
011	0.005	0.000	-1.363	3514.27	0.92	0.08
100	0.020	0.025	0.026	0.49	0.94	0.06
110	0.002	0.000	-1.082	13327.13	0.95	0.05
111	0.280	0.005	0.012	0.10	0.80	0.20

Ridge penalty						
Haplotype	Frequency	Realized^*a*^	Mean bias	Mean SE	Coverage	*P *< 0.05^*b*^

001	0.050	1.151	-0.012	0.24	0.84	0.99
101	0.003	0.817	-0.568	1.77	0.57	0.08
010	0.020	0.031	0.057	1.56	1.00	0.00
011	0.005	0.000	-0.322	2.91	0.98	0.02
100	0.020	0.025	0.022	1.65	1.00	0.00
110	0.002	0.000	-0.338	1.85	0.86	0.14
111	0.280	0.005	0.005	0.11	0.84	0.16

Difference penalty						
Haplotype	Frequency	Realized^*a*^	Mean bias	Mean SE	Coverage	*P *< 0.05^*b*^

0 0 1	0.050	1.151	0.003	0.25	0.88	0.98
1 0 1	0.003	0.817	-0.394	1.67	0.87	0.01
0 1 0	0.020	0.031	0.111	1.43	1.00	0.00
0 1 1	0.005	0.000	-0.049	2.48	0.99	0.01
1 0 0	0.020	0.025	0.078	1.53	1.00	0.00
1 1 0	0.002	0.000	-0.139	2.08	1.00	0.00
1 1 1	0.280	0.005	0.019	0.12	0.86	0.14

a) "Realized" denotes to the median estimate from the simulated data, where the haplotypes were known, and could be used in the Cox model directly; b) For haplotypes 001 and 101 these percentages reflect power, whereas the percentages for the other five haplotypes reflect type I error probabilities.

For both simulations, removal of the rare haplotypes 011, 110 and 101 from the model leads to a small reduction in the bias of the remaining haplotypes (e.g. from 0.048 to 0.038 and from 0.019 to 0.005 for haplotypes 010 and 111, respectively). Depending on the modeled effects of the omitted haplotypes, the bias in the estimate of haplotype 111 decreases (simulation 1: from 0.019 to 0.006) or increases (simulation 2: from 0.005 to 0.011) a little.

In a third simulation, replicates contained 2000 individuals and the haplotypes 001 and 101 were simulated to have a relative risk of 3 (*β *= 1.10) compared to the reference haplotype 000. This simulation mimics a setting similar to simulation 2, but due to the larger number of individuals, all 8 haplotypes will be present in all individual replicates. The mean bias, mean SE, coverage probability and percentage estimates with a p < 0.05 for this simulation are shown in Table 4.

**Table 4:** Realized *β*^*a*^, mean bias, mean standard error (SE), coverage probability and percentage of the effects that had a p-value < 0.05 in the simulation where haplotypes 001 and 101 had a modeled parameter estimate of 1.10. Each replicate contained 2000 individuals.

No penalty						
Haplotype	Frequency	Realized^*a*^	Mean bias	Mean SE	Coverage	*P *< 0.05^*b*^
001	0.050	1.094	-0.005	0.08	0.93	1.00
101	0.003	1.155	-0.037	0.38	0.93	0.77
010	0.020	0.016	-0.007	0.14	0.98	0.02
011	0.005	0.023	0.036	0.30	0.98	0.02
100	0.020	0.006	0.015	0.14	1.00	0.00
110	0.002	0.084	0.075	1.14	0.95	0.05
111	0.280	-0.007	-0.007	0.03	0.82	0.18

Ridge penalty						
Haplotype	Frequency	Realized^*a*^	Mean bias	Mean SE	Coverage	*P *< 0.05^*b*^

001	0.050	1.094	-0.009	0.07	0.91	1.00
101	0.003	1.155	-0.085	0.61	0.91	0.62
010	0.020	0.016	-0.007	0.013	0.96	0.04
011	0.005	0.023	0.046	0.23	0.96	0.04
100	0.020	0.006	0.014	0.12	0.98	0.02
110	0.002	0.084	0.067	0.40	0.66	0.34
111	0.280	-0.007	-0.008	0.03	0.81	0.19

Difference penalty						
Haplotype	Frequency	Realized^*a*^	Mean bias	Mean SE	Coverage	*P *< 0.05^*b*^

0 0 1	0.050	1.094	-0.011	0.07	0.88	1.00
1 0 1	0.003	1.155	-0.066	0.54	0.88	0.71
0 1 0	0.020	0.016	0.004	0.12	0.96	0.04
0 1 1	0.005	0.023	0.071	0.22	0.90	0.10
1 0 0	0.020	0.006	0.017	0.12	0.98	0.02
1 1 0	0.002	0.084	0.069	0.37	0.57	0.43
1 1 1	0.280	-0.007	-0.009	0.03	0.82	0.18

a) "Realized" denotes to the median estimate from the simulated data, where the haplotypes were known, and could be used in the Cox model directly; b) For haplotypes 001 and 101 these percentages reflect power, whereas the percentages for the other five haplotypes reflect type I error probabilities.

### Implementation

In the GENDER study [13] 3146 patients with cardiovascular disease who were treated with percutaneous transluminal coronary angioplasty (PTCA) with or without stents were followed for at least twelve months for the occurrence of clinical restenosis and revascularization of the vessel which was originally treated with PTCA. Inflammatory processes are involved in such target vessel revascularization (TVR), and the level of inflammatory response is controlled by several genes, among which possibly the IL10 gene. We determined in 2653 patients the variants of four SNPs in this gene (*IL10 *-592G > T, *IL10 *-2849G > A, *IL10 *-1082G > A, and *IL10 *4251A > G), and evaluated their association with TVR risk. Overall, there were 252 TVR, and TVR risk was 9% at nine months, and 10.5% at twelve months. Rare allele frequencies were 28%, 49%, 27%, and 24%, of the four SNPs, respectively. All four markers were in linkage disequilibrium (*P *< 0.001), and Hardy-Weinberg equilibrium was not rejected for any of the markers (*P *> 0.0125; significance level (Bonferroni) corrected for multiple testing).

Univariately, in a Cox model assuming co-dominant effects, *IL10 *-2849G > A, *IL10 *-1082G > A, and *IL10 *4251A > G were significantly associated with TVR risk with hazard ratios (HR) 1.21 (95% CI: 1.00–1.46), 1.20 (1.01–1.42), 1.20 (0.99–1.45), respectively. HR of *IL10 *-592C > A was 0.87 (0.70–1.07). In a Cox model with all SNPs, and all two-way interactions, we found significant interactions between SNPs *IL10 *-2849G > A, and *IL10 *-1082G > A (*P *= 0.003), and *IL10 *-1082G > A, and *IL10 *4251A > G (*P *= 0.001). Higher-order interactions were not significant. The prognostic index of this model (*Xβ*) varied between -2.6 and +1.6, and had 23 different values corresponding to the 23 different genotype combinations that were observed.

**Figure 1:**
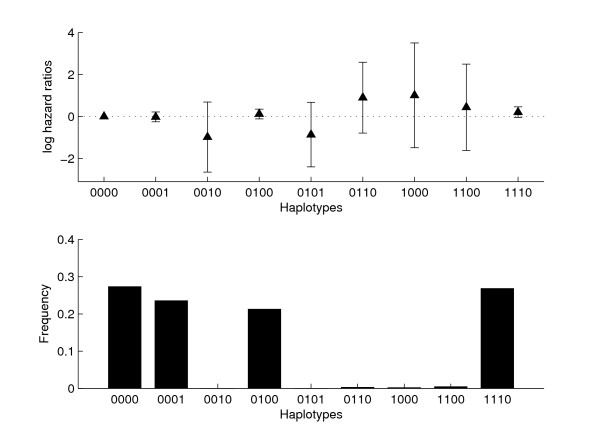
Frequencies and log TVR hazard ratios with 95% confidence intervals of the IL10 haplotypes.

With 4 biallelic SNPs, 16 different haplotypes are possible, but seven had zero frequency in the current sample. Of the remaining nine haplotypes, there were four major haplotypes with substantial frequencies 27% (0000), 24% (0001), 21% (0100), and 27% (1110), while the relative frequencies of the remaining five haplotypes varied between 0.46% and 0.02%. The estimated haplotype frequencies and log hazard ratios at the optimal CVL (ridge penalty) are given in Figure 1. The hazard ratios of the four major haplotypes were not different from each other, but of the five rare haplotypes two had decreased (0010, and 0101), and three had increased (0110, 1000, and 1100) hazard ratios, although all had very wide confidence intervals, and none were statistically significantly associated with TVR risk.

**Figure 2:**
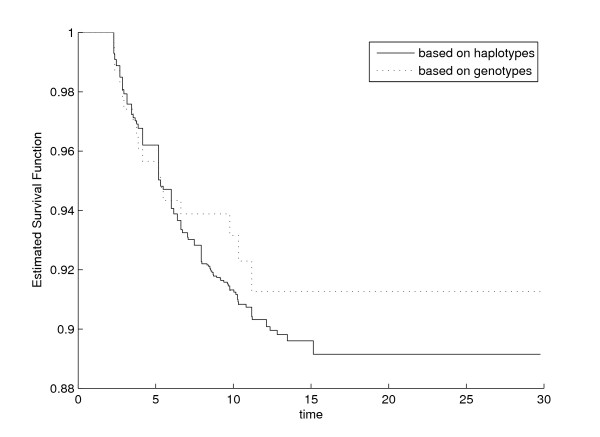
Estimated IL10 survival curves based on genotypes and based on haplotypes.

To validate our model we calculated the survival curves of all 23 subgroups with different genotype combinations according to equation (3) and compared these with the Kaplan-Meier curves. These curves are given in Figure 2 for the subgroup of 233 patients who were homozygous for the most common allele of *IL10 *-2849G > A and *IL10 *4251A > G, and heterozygous for *IL10 *-1082G > A and *IL10 *-592C > A (Figure 2). These two curves are comparable, indicating that our haplotype method produces valid results.

## Discussion

In the present study we present a method to model the relation between failure time and (rare) haplotypes in unrelated individuals. The simulations presented in this study show that haplotype effects of especially the rare haplotypes are closer to the true estimates when a penalty is introduced into the model.

Furthermore, the simulations show that the penalized log-likelihood approach that is used to deal with the unstable estimates of rare haplotypes can indeed shrink the estimates and their 95% confidence intervals to 'acceptable' values. Simulations also show that the cross-validated standard errors of the more common haplotypes can be increased compared to their unpenalized standard errors due to uncertainty with respect to the penalty parameter *λ*. The power to detect a true haplotype effect is, in general, reduced in the penalized models compared to the non-penalized model, the reduction being more pronounced for the less frequent haplotypes. This reduced power is due to the shrinkage of the estimates. With respect to the type I error probabilities, the effect of introducing a penalty depends on the penalty applied and the true haplotype effects. For the ridge penalized models, the type I error probabilities are similar to those observed in the non-penalized models. The non-penalized estimates of the haplotypes without a modeled effect are already close to the true value of zero and the nature of the ridge penalty further shrinks the effects towards zero. For the difference penalty, the type I error probability appears to be increased for some haplotypes. This deviation is related to the extent that the assumption (similar haplotypes, similar effects) is met. In the first simulation (Table 2), the mean bias of haplotypes 001 and 100 are increased in the direction of a *β *> 0. In this scenario, haplotypes 010, 011, 110 and 111 all had a modeled effect and the difference penalty results in estimates for the haplotypes 001 and 100 towards the effects of these haplotypes. In the second simulation, the majority of the haplotypes had no modeled effect and the effects of the rare haplotypes could be directed towards *β *= 0. In replicates containing 2000 individuals (Table 4), the reduction in SE is still present, but the gain is relatively small compared to simulation with only 200 individuals per replicate. This is conform expectations, since rare haplotypes are 'less rare' in larger samples, thus enabling a more precise estimation of their effect even without a penalty. Based on the characteristics of the models displayed in the simulations and the real data, the penalized log-likelihood method mostly serves the purpose of estimating the effects of rare haplotypes more accurate.

The method described in this paper is a flexible method allowing for adjustment for (environmental) covariates as well as haplotype-environment interactions. Although we focus on haplotypes consisting of a certain number of biallelic SNPs, the method is also capable to handle loci with more than two alleles. Furthermore, the method can be easily extended to deal with missing genotype data, since this will simply increase the number of possible haplotype pairs that are compatible with the observed genotype. The *wiq *in our method are calculated under the assumption of Hardy-Weinberg. Although we did not check robustness of the method to violations of this assumption, Lin [10] has shown that his method, which is similar to our unpenalized method, is robust to violations of the Hardy-Weinberg assumption.

As an alternative estimation method we considered the partial likelihood. Unfortunately, estimation of *β *and *H*_0_(*t*) are not separated, and therefore there is no reason to prefer this partial likelihood approach over the EM algorithm outlined in the present manuscript. Compared to the method described by Tregouet et al [11], the present EM method assumes piecewise constant hazard, which seems less restrictive than the assumption behind the their method using partial likelihood.

We use a penalty function to increase precision of estimates of rare haplotypes. Other strategies for managing unstable estimates of rare haplotypes include excluding the rare haplotypes from the variable list, pooling the rare haplotypes into one category, or pooling the rare haplotypes with common haplotypes that are very similar. The first approach implicitly groups the rare haplotypes with the reference category and the second and third approach lead to pooled categories that are sometimes hard to interpret. Nevertheless, these last two methods seem to increase power [16]. However, the three strategies mentioned above do not result in (individual) effect estimates of rare haplotypes, whereas the penalized models do.

## Conclusion

The method presented in this paper can be applied to estimate haplotype effects in cohort studies when haplotype phase is unknown. The joint estimation of haplotype effects and haplotype frequencies together with the penalty function provides a good way of estimating effects of rare haplotypes, which is a common problem in these studies.

## Abbreviations

SNP(s): single nucleotide polymorphism(s); SE: standard error; PTCA: percutaneous transluminal coronary angioplasty; TVR: target vessel revascularization; IL10: interleukin 10.

## Authors' contributions

OWS participated in programming the method, performed the statistical analysis of the data and drafted the manuscript. AHZ was responsible for the theoretical background of the method, participated in the programming of the method and the analysis of the data and helped to draft the manuscript. JWJ was responsible for the collection of the data and critically evaluated the manuscript. MWTT participated in the development of the method, performed the simulations and helped to draft the manuscript. All authors read and approved the final manuscript.
